# Supplementary material for: Modeling glioblastoma heterogeneity as a dynamic network of cell states
Source: Mol Syst Biol. 2021 Sep 16;17(9):e10105. doi: 10.15252/msb.202010105 (PMC8444284; doi:10.15252/msb.202010105)
Supplement: Supplementary file 5 — Source Data for Figure 3 [file MSB-17-e10105-s001.zip › Figure3A_sourcedata/GSEA_3065/hallmarks_state6.GseaPreranked.1623416308702/HALLMARK_REACTIVE_OXYGEN_SPECIES_PATHWAY.html]

Details for gene set HALLMARK\_REACTIVE\_OXYGEN\_SPECIES\_PATHWAY[GSEA]

|  || Dataset | state6 |
| Phenotype | NoPhenotypeAvailable |
| Upregulated in class | na\_pos |
| GeneSet | HALLMARK\_REACTIVE\_OXYGEN\_SPECIES\_PATHWAY |
| Enrichment Score (ES) | 0.2891144 |
| Normalized Enrichment Score (NES) | 1.0048604 |
| Nominal p-value | 0.44067797 |
| FDR q-value | 0.5839606 |
| FWER p-Value | 1.0 |
Table: GSEA Results Summary

  

Fig 1: Enrichment plot: HALLMARK\_REACTIVE\_OXYGEN\_SPECIES\_PATHWAY      
 Profile of the Running ES Score & Positions of GeneSet Members on the Rank Ordered List

  

| PROBE | GENE SYMBOL | GENE\_TITLE | RANK IN GENE LIST | RANK METRIC SCORE | RUNNING ES | CORE ENRICHMENT || 1 | FTL |  |  | 64 | 0.234 | 0.0820 | Yes |
| 2 | ATOX1 |  |  | 179 | 0.183 | 0.1386 | Yes |
| 3 | GPX4 |  |  | 365 | 0.141 | 0.1711 | Yes |
| 4 | STK25 |  |  | 412 | 0.134 | 0.2170 | Yes |
| 5 | NDUFA6 |  |  | 730 | 0.103 | 0.2201 | Yes |
| 6 | LAMTOR5 |  |  | 949 | 0.088 | 0.2289 | Yes |
| 7 | GLRX |  |  | 1028 | 0.084 | 0.2521 | Yes |
| 8 | PRDX2 |  |  | 1061 | 0.082 | 0.2797 | Yes |
| 9 | SOD2 |  |  | 1228 | 0.075 | 0.2891 | Yes |
| 10 | PTPA |  |  | 1697 | 0.057 | 0.2573 | No |
| 11 | PFKP |  |  | 2583 | 0.031 | 0.1682 | No |
| 12 | TXNRD2 |  |  | 2654 | 0.030 | 0.1716 | No |
| 13 | GLRX2 |  |  | 3010 | 0.022 | 0.1394 | No |
| 14 | NDUFB4 |  |  | 3026 | 0.021 | 0.1459 | No |
| 15 | OXSR1 |  |  | 3182 | 0.018 | 0.1351 | No |
| 16 | SBNO2 |  |  | 3223 | 0.017 | 0.1371 | No |
| 17 | TXNRD1 |  |  | 3380 | 0.014 | 0.1244 | No |
| 18 | GSR |  |  | 3436 | 0.012 | 0.1228 | No |
| 19 | SCAF4 |  |  | 3664 | 0.008 | 0.0998 | No |
| 20 | ERCC2 |  |  | 3957 | 0.001 | 0.0670 | No |
| 21 | CAT |  |  | 3983 | 0.001 | 0.0645 | No |
| 22 | HMOX2 |  |  | 3988 | 0.001 | 0.0643 | No |
| 23 | JUNB |  |  | 4044 | -0.000 | 0.0581 | No |
| 24 | CDKN2D |  |  | 4667 | -0.012 | -0.0084 | No |
| 25 | PDLIM1 |  |  | 5379 | -0.026 | -0.0797 | No |
| 26 | G6PD |  |  | 5387 | -0.026 | -0.0706 | No |
| 27 | PRDX1 |  |  | 5508 | -0.029 | -0.0734 | No |
| 28 | GCLM |  |  | 5804 | -0.034 | -0.0940 | No |
| 29 | NDUFS2 |  |  | 5981 | -0.038 | -0.0997 | No |
| 30 | TXN |  |  | 6726 | -0.057 | -0.1631 | No |
| 31 | SOD1 |  |  | 7066 | -0.067 | -0.1764 | No |
| 32 | PRDX6 |  |  | 7589 | -0.085 | -0.2035 | No |
| 33 | PRNP |  |  | 8140 | -0.117 | -0.2217 | No |
| 34 | SELENOS |  |  | 8258 | -0.127 | -0.1868 | No |
| 35 | MSRA |  |  | 8546 | -0.172 | -0.1542 | No |
| 36 | PRDX4 |  |  | 8660 | -0.217 | -0.0845 | No |
| 37 | NQO1 |  |  | 8725 | -0.262 | 0.0081 | No |
Table: GSEA details [plain text format]

  

Fig 2: HALLMARK\_REACTIVE\_OXYGEN\_SPECIES\_PATHWAY: Random ES distribution      
 Gene set null distribution of ES for **HALLMARK\_REACTIVE\_OXYGEN\_SPECIES\_PATHWAY**
